# Diagnostic performance of the normal range of gastrin calculated using strict criteria based on a combination of serum markers and pathological evaluation for detecting gastritis: a retrospective study

**DOI:** 10.1186/s12876-023-02816-1

**Published:** 2023-05-20

**Authors:** Naoko Nagasaki, Hidehiko Takigawa, Masanori Ito, Tomoyuki Boda, Takahiro Kotachi, Ryohei Hayashi, Ryo Yuge, Yuji Urabe, Shiro Oka, Shinji Tanaka

**Affiliations:** 1 Department of Gastroenterology, Hiroshima University Hospital, Hiroshima, Japan; 2 Department of Endoscopy, Hiroshima University Hospital, 1-2-3 Kasumi, Minami-Ku, Hiroshima-Shi, Hiroshima, 734-8553 Japan; 3 Department of General Internal Medicine, Hiroshima University Hospital, Hiroshima, Japan; 4 Department of Internal Medicine, Hiroshima Memorial Hospital, Hiroshima, Japan; 5 Division of Regeneration and Medicine Center for Translational and Clinical Research, Hiroshima University Hospital, Hiroshima, Japan

**Keywords:** Gastrin, Gastritis, *Helicobacter pylori*, Gastric cancer, Normal range

## Abstract

**Background:**

The ABC method, which combines the pepsinogen method and anti*-Helicobacter pylori* antibody titers, has been used for risk screening for gastric cancer in Japan. However, it has been reported that there are cases of gastritis and carcinogenesis risk even in group A, which is considered to be a low-risk group based on the ABC method. Currently, in group A, endoscopic examination is needed to strictly discriminate “patients without gastritis” (defined as true A patients) from those “with gastritis.” A simple and minimally invasive diagnostic criterion for gastritis using serological markers is desirable. In this study, we aimed to identify the normal serum gastrin concentrations in normal stomach cases based on pathological diagnosis and investigate the usefulness of serum gastrin concentrations in diagnosing gastritis.

**Methods:**

Patients who underwent endoscopy and blood tests at Hiroshima University Hospital were enrolled in the study and categorized into the “pathologically-evaluated group” and “endoscopically-evaluated group,” according to the evaluation method of atrophic gastritis. Initially, we measured serum gastrin concentrations in the normal stomach cases in the pathologically-evaluated group and calculated the normal range of serum gastrin concentrations. We used the upper limit of this normal range of serum gastrin concentrations and performed a validation study to determine its usefulness as a diagnostic marker for distinguishing between cases of gastritis and true A in the endoscopically-evaluated group.

**Results:**

The 95th percentile of serum gastrin concentrations in pathologically-evaluated normal stomach cases was 34.12–126.03 pg/mL. Using the upper limit of this normal range of serum gastrin concentrations, the sensitivity, specificity, positive predictive value, and negative predictive value for gastritis were 52.8%, 92.6%, 97.0%, and 31.0%, respectively. Additionally, the receiver operating characteristic (ROC) curve for the endoscopically-evaluated group showed an area under the ROC curve of 0.80.

**Conclusion:**

The gastrin cut-off value of 126 pg/mL has a good positive predictive value (97.0%) for detecting gastritis positing its use as a marker for cases requiring endoscopy. However, the identification of patients with gastritis having normal serum gastrin concentrations due to insufficient sensitivity remains a challenge for the future.

**Supplementary Information:**

The online version contains supplementary material available at 10.1186/s12876-023-02816-1.

## Background

Gastric cancer is the fifth most common cancer worldwide and the fourth leading cause of cancer deaths. Japan has one of the highest incidence rates of gastric cancer [1]. Numerous epidemiological and clinical studies have shown that *Helicobacter pylori* (*Hp*) infection is an important and essential factor in the development of gastric cancer [2–6]. In Japan, the prevalence of true *Hp*-negative gastric cancer is extremely low [7, 8]. Therefore, there is a need for a simple and minimally invasive method for diagnosing *Hp* infection to screen for gastric cancer. It has been reported that *Hp* infection-related atrophic gastritis is a risk factor for gastric cancer [9–11]. Furthermore, the presence of serum pepsinogen (PG) has been reported to reflect extensive atrophic gastritis; hence, a serum screening system using PG concentrations has been developed [12, 13]. Subsequently, the ABC method—combining serum anti-*Hp* antibody (Ab) titers and serum PG concentrations (serum PG I concentration or PG I/II ratio)—was shown to be effective in assessing the risk of gastric cancer. According to the ABC method, the risk for gastric cancer can be stratified into four groups: group A, PG negative and anti-*Hp* Ab negative; group B, PG negative and anti-*Hp* Ab positive; group C, PG positive and anti-*Hp* Ab positive; and group D, PG positive and anti-*Hp* Ab negative [14].

It has been reported that when group A patients were classified into the “true A group” (those who did not have endoscopic atrophy) and “pseudo-A group” (those with endoscopic atrophy) based on endoscopy, the former demonstrated extremely low carcinogenicity [15–17], whereas the latter demonstrated high carcinogenicity [18, 19]. Therefore, it is important to take endoscopic findings into account in the ABC method to discriminate true A patients from pseudo-A patients in the assessment of gastric cancer risk [7].

GastroPanel is a non-invasive tool for the diagnosis of atrophic gastritis; it combines serological assays of PG, gastrin, and anti-*Hp* Abs [20–23]. Gastrin is included in GastroPanel to evaluate the gastric mucosa. Several studies have reported on serum gastrin concentrations in gastritis cases [24, 25] and on the fact that gastrin concentrations are elevated in gastritis and can be a marker of gastritis [25, 26]; however, the gastrin concentrations in these studies were measured without prior pathological evaluation. To the best of our knowledge, there are no reports on the normal gastrin concentrations in the true A group.

The reference range of gastrin concentrations is set to < 200 pg/mL in commercial kits, which is based on a mean value of 87.8 pg/mL and a standard deviation of 38.4 pg/mL, derived from a series of studies on fasting blood gastrin concentrations in 129 healthy individuals from three research institutions in Japan [27, 28]. However, in these studies, 200 pg/mL is not considered an adequate cut-off value for a pathologically normal stomach.

Therefore, in this study, we aimed to establish the normal serum concentrations of gastrin in normal stomach cases, according to strict criteria based on the combination of the ABC method and pathological diagnosis as per the Updated Sydney System (USS) [29].

Furthermore, we evaluated the validity of this gastrin cut-off value in diagnosing gastritis in a separate group that was evaluated endoscopically.

## Methods

### Study design and patients

This retrospective study evaluated 14,788 patients who underwent upper gastrointestinal endoscopy and serum marker evaluation (PG I, PG II, PG I/II ratio, gastrin, and anti-*Hp* Abs) at Hiroshima University Hospital, Japan, between 1990 and 2014. The exclusion criteria were as follows: patients with missing laboratory values; patients with esophagitis, peptic ulcers, or upper gastrointestinal neoplasms; patients on gastric acid secretion inhibitors; patients having undergone successful eradication therapy; patients who had undergone gastrectomy; patients with severe liver or kidney dysfunction; those diagnosed with Zollinger–Ellison syndrome; and those diagnosed with autoimmune gastritis. First, a pathologically-evaluated group of 467 patients (mean age: 49.7 years [range, 16 − 88 years], 268 males and 199 females), whose presence or absence of gastritis could be determined by pathological examination, were included. Gastrointestinal endoscopy records of 8448 matched patients from the Hiroshima Prefectural Regional Cancer Registry database were checked. In total, 1317 patients who could be followed up for at least 1 year with annual endoscopy were then selected as an endoscopically-evaluated group (mean age: 62.3 years [range, 14 − 95 years], 860 males and 457 females). The flow diagram for the selection process of the study is presented in Supplementary Fig. 1, according to the Standards for Reporting of Diagnostic Accuracy Studies [30]. This study was approved by the Institutional Review Board of Hiroshima University Hospital (approval number E-4237) and was conducted in accordance with the tenets of the Declaration of Helsinki. The need for informed consent was waived owing to the use of anonymized data. However, we used the opt-out method for participation in the study.

### Evaluation of serum markers

Fasting blood samples were collected, and serum samples were stored at –20 °C until further use for both groups. Serum gastrin concentrations were measured using the Gastrin RIA Kit II (Dynabot, Tokyo, Japan). Serum PG I and PG II concentrations were determined by radioimmunoassay (Abbott, Tokyo, Japan) for the years 1990 to 1999, chemiluminescent immunoassay (Abbott) from 1999 to 2001, enzyme immunoassay (E-plate test; Eiken, Tokyo, Japan) from 2001 to 2003, and latex agglutination test (L-Z test; Eiken) from 2003 to 2014. PG positivity was defined as serum PG I concentrations of ≤ 70 ng/mL and a PG I/II ratio of ≤ 3.0 [31–33]. Serum anti-*Hp* Abs in the pathology group was determined with the Pyloristat kit (Whittaker Bioproducts, Walkerville, MD, USA) using enzyme-linked immunosorbent assay (ELISA). Serum anti-*Hp* Abs titers of the endoscopically-evaluated group were evaluated using ELISA (E-plate; Eiken Chemical). The cut-off value for anti-*Hp* Abs in the endoscopically-evaluated group was 3 U/mL [34, 35].

### Evaluation of gastritis in the pathologically-evaluated group

Four biopsies were taken from four sites within each patient: two from the antrum of the stomach and two from the corpus of the stomach [36]. Biopsy specimens were immediately fixed in 10% formalin, embedded in paraffin, cut into 4-μm sections, and subjected to hematoxylin and eosin staining and Giemsa staining. Two pathologists, blinded to serological data, evaluated all biopsy specimens to determine *Hp* infection, mucosal inflammation, neutrophil activity, glandular atrophy, and intestinal metaplasia based on the USS criteria. Five items were graded on a 4-point scale, from 0 to 3 (0: none, 1: mild, 2: moderate, and 3: severe). Histopathological findings were categorized into Grade 0 (no findings at all) and Grade 1 − 3 (presence of some findings) [37]. Diagnostic disagreements were resolved by collaborative discussion. Gastritis was determined based on pathological and serological evaluation. Specifically, normal stomach was defined as those cases that were deemed to be Grade 0 for all gastritis indices as per USS criteria and were negative for anti-*Hp* Ab and PG (Supplementary Table 1 in Additional file 2).

### Evaluation of gastritis in the endoscopically-evaluated group

In the endoscopically-evaluated group, we conducted a validation study for the diagnosis of gastritis based on the upper limit of the normal range of serum gastrin concentrations calculated in the pathologically-evaluated group.

Atrophic gastritis status was evaluated based on the Kimura–Takemoto classification. Absence of atrophic gastritis was defined as “no endoscopic atrophic changes in the gastric body corresponding to C-0 or C-1 in the Updated Kimura–Takemoto classification” [38]. Group A cases with endoscopic atrophy were defined as the “pseudo-A group,” and this group together with groups B, C, and D, were classified as “others” (Supplementary Table 1 in Additional file 2).

### Statistical analysis

Data are expressed as mean ± standard deviation (SD). Categorical data were compared using the χ^2^-test and Fisher’s exact test as appropriate. Wilcoxon rank-sum tests were used for categorical data. The diagnostic accuracy for “true A” in the endoscopically-evaluated group was evaluated using receiver operating characteristic (ROC) curve analysis. All statistical analyses were performed using the JMP statistical software (SAS Institute Inc., Cary, NC, USA).* P* < 0.05 was considered statistically significant.

## Results

### Characteristics of the pathologically-evaluated group

The mean ± SD age and serum gastrin concentrations of patients in the pathologically-evaluated group was 49.7 ± 18.4 years and 159.74 ± 124.41 pg/mL, respectively. Of the 467 patients in this group, 327 were positive for anti-*Hp* Abs. The mean ± SD serum PG I concentration was 43.7 ± 21.5 ng/mL, and the mean ± SD serum PG I/II ratio was 3.57 ± 1.96. Table 1 shows the baseline characteristics of the 467 patients in the pathology group. According to the pathological examination based on the USS, 105, 108, 153, 355, and 125 patients were determined to be Grade 0 for the gastritis indices of neutrophil activity, inflammation, atrophy, intestinal metaplasia, and *H. pylori* infection, respectively. Ninety-six of the 467 patients were pathologically diagnosed to be normal stomach cases based on the following factors: (i) negative serum anti-*Hp* Abs, (ii) negative PG, (iii) no gastritis on endoscopy, and (iv) Grade 0 for all pathological gastritis indices according to the USS. In the pathologically-evaluated group, the distribution of participants with negative/positive serum anti-*Hp* Abs, negative/positive PG, and presence/absence of gastritis based on the USS is shown in Supplementary Table 2 in Additional file 2. Of the 131 patients with negative serum anti-*Hp* Abs and negative PG, 35 patients (35/131, 27%) had gastritis based on the USS. Furthermore, patients who were positive for either serum anti-*Hp* Abs or PG were evaluated as having gastritis based on the USS.

**Table 1:** Baseline characteristics of the pathologically-evaluated group and comparison of the pathologically-evaluated normal stomach and others groups

Characteristics		Total (*n* = 467)	Pathologically-evaluated normal stomach (*n* = 96)	Others (*n* = 371)	*P*-value
Age (years), mean ± SD		49.7 ± 18.4 (16–88)	35.1 ± 15.3	53.4 ± 17.2	< 0.05
Sex (male/female)		268/199	56/40	212/159	0.83
PG I (ng/mL)		43.7 ± 21.5	39.5 ± 12.4	44.8 ± 23.2	< 0.05
PG II (ng/mL)		14.9 ± 8.64	6.67 ± 2.35	17.08 ± 8.37	< 0.05
PG I/II ratio		3.57 ± 1.96	6.13 ± 1.26	2.90 ± 1.50	< 0.05
Gastrin (pg/mL)		159.74 ± 124.41	70.9 ± 27.8	182.5 ± 146.6	< 0.05
Anti-*Hp* Ab	Positive	327	0	327	< 0.05
	Negative	140	96	44	
Endoscopic atrophy (negative/positive)		120/347	96/0	24/347	< 0.05
Histologic gastritis (Grade 0/1–3)^※^	Neutrophil activity	105/362	96/0	9/362	< 0.05
	Inflammation	108/359	96/0	12/359	< 0.05
	Atrophy	153/314	96/0	57/314	< 0.05
	Intestinal metaplasia	355/112	96/0	259/112	< 0.05
	*Hp* infection	125/342	96/0	29/342	< 0.05

※Grade 0–4 (0, none; 1, mild; 2, moderate; 3, severe)

*anti-Hp Ab* anti-*Hp* antibody, *PG* Pepsinogen, *Hp Helicobacter pylori*, *SD* Standard deviation

### Comparison between normal stomach and others in the pathologically-evaluated group

**Fig. 1:**
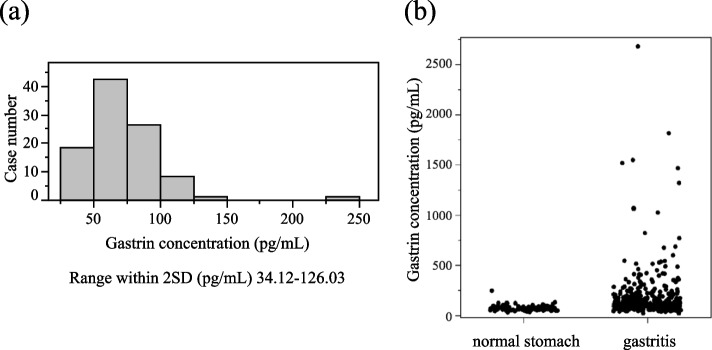
Number of subjects in the pathologically-evaluated normal stomach group. **a** Histogram showing the trends in serum gastrin concentrations in the pathologically-evaluated normal stomach group. **b** Comparative distribution of serum gastrin concentrations between the gastritis and normal stomach subgroups in the pathologically-evaluated group. SD, standard deviation

The pathologically-evaluated group included 96 patients without gastritis and 371 patients with gastritis (Table 1). The patients in the normal stomach group were younger than those in the gastritis group (mean ± SD age: 35.1 ± 15.3 *vs.* 53.4 ± 17.2 years, respectively) and had lower mean ± SD serum PG I concentrations (39.5 ± 12.4 *vs.* 44.8 ± 23.2 ng/mL, respectively) and serum PG II concentrations (6.67 ± 2.35 *vs*. 17.08 ± 8.37 ng/mL, respectively). However, patients in the normal stomach group had a significantly higher serum PG I/II ratio compared to those in the gastritis group (mean ± SD: 6.13 ± 1.26 *vs.* 2.90 ± 1.50, respectively; *P* < 0.05) and a significantly lower serum gastrin concentration (mean ± SD: 70.9 ± 27.8 *vs.* 182.5 ± 146.6 pg/mL, respectively) (Fig. 1b). Serum gastrin concentrations ranged from 29 pg/mL to 243 pg/mL in the normal stomach group, and the 95^th^ percentile of normal ranged from 34.12 pg/mL to 126.03 pg/mL (Fig. 1a). At the upper limit of normal, 126.03 pg/mL was identified as the cut-off value.

Subsequently, we conducted a validation study to verify the possibility that the normal serum gastrin concentrations measured from the pathologically-evaluated group could be used as a marker to distinguish gastritis cases from *Hp*-uninfected cases in the endoscopically-evaluated group.

### Patient characteristics in the endoscopically-evaluated group

Table 2 shows the baseline characteristics of the 1317 patients in the endoscopically-evaluated group. The mean ± SD age at baseline was 62.3 ± 13.7 years, and these patients were approximately 10 years older than those in the pathologically-evaluated group. The mean ± SD serum gastrin concentration was 198.26 ± 136.47 pg/mL. There were 323, 452, 474, and 68 patients in groups A, B, C, and D, respectively. Among group A patients, 243 and 80 patients were classified as “true A” and “pseudo-A,” respectively. The mean ± SD serum PG I concentration was 56.9 ± 40.7 ng/mL, and the mean ± SD serum PG I/II ratio was 3.39 ± 1.98. Additionally, 289 patients did not have endoscopic gastric mucosal atrophy, 208 patients had closed type endoscopic gastric mucosal atrophy, and 820 patients had open type endoscopic gastric mucosal atrophy. Twenty-one patients developed cancer at follow-up evaluation.

**Table 2:** Baseline characteristics of the endoscopically-evaluated group

Characteristics		Values
Age (years), mean ± SD (range)		62.3 ± 13.7 (14–95)
Sex (M/F)		860/457
ABC method	True A	243
	Pseudo A	80
	B	452
	C	474
	D	68
Gastrin (pg/mL), mean ± SD		198.26 ± 136.47
PG I (ng/mL), mean ± SD		56.9 ± 40.7
PG II (ng/mL), mean ± SD		19.4 ± 14.4
PG I/II ratio, mean ± SD		3.39 ± 1.98
Endoscopic atrophy	None	289
	Closed	208
	Open	820
Patients with cancer		21

*PG* Pepsinogen, *SD* Standard deviation

### Comparison between true A and others in the endoscopically-evaluated group

The 1317 patients in the endoscopically-evaluated group were categorized into the true A group (*n* = 243) and the “others” group (which included the pseudo-A group as well as group B, group C, and group D as per the ABC method; *n* = 1074) (Table 3). The mean ± SD age of patients in the true A group was 53.2 ± 16.3 years. There were more male patients in the others group than in the true A group (male: female ratio, 2:1 *vs.* 3:2, respectively). For serum PG concentrations, the PG I concentration and PG I/II ratio were significantly higher in the true A group than in the others group. Meanwhile, the serum gastrin concentration was significantly lower in the true A group than in the others group (mean ± SD: 75.0 ± 33.3 *vs.* 226.2 ± 153.2 pg/mL, respectively). The true A group had no cases of endoscopic atrophy, whereas 96% of the patients in the others group had endoscopic atrophy. All patients who developed cancer belonged to the others group.

**Table 3:** Comparison between true A and others groups in the endoscopically-evaluated group

Characteristics		True A (*n* = 243)	Others (*n* = 1074)	*P*-value
Age (years), mean ± SD		53.2 ± 16.3	64.3 ± 12.1	< 0.05
Sex (male/female)		142/101	718/356	< 0.05
Gastrin (pg/mL)		75.0 ± 33.3	226.2 ± 153.2	< 0.05
PG I (ng/mL)		70.6 ± 39.7	53.7 ± 40.3	< 0.05
PG II (ng/mL)		13.1 ± 11.9	20.8 ± 14.5	< 0.05
PG I/II ratio		5.74 ± 1.38	2.85 ± 1.69	< 0.05
Endoscopic atrophy	(–)	243	46	< 0.05
	( +)	0	1028	
Patients with cancer		0	21	< 0.05

*PG* Pepsinogen, *SD* Standard deviation

Validation of serum gastrin concentrations and comparison between patients with high and normal gastrin concentrations in the endoscopically-evaluated group.

**Fig. 2:**
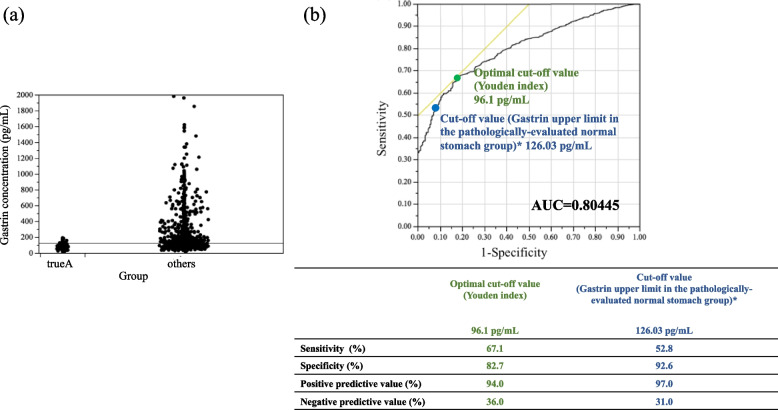
Validation of the diagnostic performance of the gastrin cut-off value in the endoscopically-evaluated group. **a** Comparative distribution of serum gastrin concentrations between the true A and “others” subgroups in the endoscopically-evaluated group. The horizontal line indicates the cut-off value, 126.03 pg/mL, which is the upper limit of the 95^th^ percentile of the normal range of serum gastrin concentrations calculated in the pathologically-evaluated normal stomach group. **b** Receiver operating characteristic (ROC) curves with the cut-off value for detecting gastritis in the endoscopically-evaluated group. Area under the ROC curve = 0.80

We evaluated the diagnostic performance of normal serum concentrations of gastrin as determined from laboratory values for normal stomach patients, who were diagnosed as such by pathological evaluation. This normal concentration of gastrin was then used to distinguish patients with atrophic gastritis from true A in the endoscopically-evaluated group (Fig. 2a). The optimal cut-off value of gastrin, according to the Youden index, was 96 pg/mL. The cut-off value based on the upper limit of the serum concentration of gastrin in normal stomach cases in the pathologically-evaluated group was 126.03 pg/mL. The sensitivity, specificity, positive predictive value, and negative predictive value for gastritis at the cut-off value of 126.03 pg/mL were 52.8%, 92.6%, 97.0%, and 31.0%, respectively. In addition, ROC curves showed high diagnostic performance of gastrin for gastritis, with an area under the ROC (AUC) curve of 0.80 (Fig. 2b).

Patients with gastrin concentrations ≤ 126.03 pg/mL (cut-off value) were determined to have normal gastrin; these patients were significantly younger and included a higher proportion of true A patients than high gastrin group. These patients also had significantly lower serum PG II concentrations and a significantly higher PG I concentration and PG I/II ratio. There was no significant difference in the number of patients who developed cancer at follow-up between the high and normal gastrin groups (Table 4).

**Table 4:** Comparison between the high and normal gastrin groups in the endoscopically-evaluated group

Characteristics		Normal gastrin group (≤ 126.03 pg/mL)	High gastrin group (> 126.03 pg/mL)	*P*-value
		*n* = 732	*n* = 585	
Age (years), mean ± SD		59.4 ± 14.6	65.8 ± 11.7	< 0.05
Group, N (%)	True A	225 (31)	18 (3)	< 0.05
	Others	507 (69)	567 (97)	
PG I (ng/mL)		58.9 ± 36.7	54.3 ± 45.1	< 0.05
PG II (ng/mL)		16.0 ± 9.93	23.7 ± 17.6	< 0.05
PG I/II ratio		4.14 ± 1.88	2.44 ± 1.68	< 0.05
Cancer patients, N (%)		12 (1.64)	9 (1.54)	0.88

*PG* Pepsinogen, *SD* Standard deviation

## Discussion

In this study, the normal concentration of serum gastrin was calculated in patients with pathologically confirmed normal stomach based on strict evaluation criteria. By applying this gastrin cut-off value to other groups, we demonstrated that the calculated normal gastrin concentration could distinguish patients with gastritis from those with normal stomach with high specificity (92.6%) and a high positive predictive value (97.0%). We therefore propose that serum gastrin concentration is a good indicator for recommending endoscopy.

Importantly, the serum gastrin concentration evaluated in this study showed high diagnostic performance for identifying patients with gastritis in the endoscopically-evaluated group. Although, the specificity and AUC were high, the sensitivity was normal. Therefore, even when the gastrin concentration is low, the possibility of gastritis cannot be ruled out. However, if the gastrin concentration is above the cut-off value, the possibility of gastritis is high. In such cases, we recommend endoscopic examination. It may be difficult to use as screening test, but it may serve as an effective diagnostic tool in recommending endoscopy to patients who are reluctant to undergo endoscopy.

In Western countries, GastroPanel is used for serological screening. However, its reliability remains unclear despite various reports [39–42]. Serum PG I, PG II, PG I/II ratio, and gastrin have been reported as potential serological biomarkers to screen for atrophic gastritis in Iran, a country with a high incidence of gastric cancer [43]. A meta-analysis also reported that a combination of serum PG, gastrin, and anti-*Hp* Abs is useful in the diagnosis of gastritis [44]. However, some studies have concluded that GastroPanel is not useful [41, 42]. The diagnostic performance of GastroPanel for gastritis was higher than that of PG I alone, although the difference was not statistically significant [42]. In this study, the comparison of true A and others in the endoscopically-evaluated group showed no discrepancy with the ABC method, as both serum PG I and PG I/II ratio were decreased in the gastritis cases. The combination of serum PG, gastrin, and anti-*Hp* Abs was useful for diagnosis of gastritis. There are many reports on the PG method and anti-*Hp* Abs in Japan; however, there are few reports on gastrin as a screening method for atrophic gastritis, thus making our study valuable.

In this study, we retrospectively reviewed cases where tissue specimens had been previously collected. It is difficult to prospectively collect a large number of patients for diagnosis by biopsy using the USS because of the invasiveness and cost of the procedure. The significance of this study is that we identified true normal stomach cases and calculated the normal range of serum gastrin concentrations by combining the PG method and anti-*Hp* Abs in normal stomach cases, using a group of 462 pathologically diagnosed cases. Of note, validation was performed using gastrin concentrations measured by the same assay method in two large groups, the pathologically-evaluated and endoscopically-evaluated groups.

One limitation of this study is that the method of measuring serum anti-*Hp* Abs and PG concentrations differed depending on the year, as these assays change yearly in clinical practice. And we showed the usefulness of a serum gastrin cut-off value focusing on distinguishing gastritis cases from normal stomach cases in this study. However, the cut-off value to distinguish true A from pseudo-A was not analyzed in this study. This is also an important point and an issue to address in future studies.

## Conclusion

The gastrin cut-off value of 126 pg/mL has a good positive predictive value (97.0%) for detecting gastritis, suggesting that it can be used as a marker for recommending endoscopy. However, it is noteworthy that some patients with gastritis do not have high serum gastrin concentrations. The identification of these patients remains a challenge for the future.

## Supplementary Information

**Additional file 1**: **Supplementary Fig. 1.** Analysis flow.

**Additional file 2**: **Supplementary Table 1.** Definitions of the pathologically- and endoscopically- evaluated groups. **Supplementary table 2.** Comparison between classification by the ABC methods and by Updated Sydney System for diagnosis of gastritis.

## Data Availability

All data generated or analyzed during this study are included in this published article and its supplementary information files.

## Abbreviations

PG: Pepsinogen
ROC: Receiver operating characteristic
ELISA: Enzyme-linked immunosorbent assay
USS: Updated Sydney system
*Hp*: *Helicobacter pylori*
anti-*Hp* Ab: Anti-*Hp* antibody
AUC: Area under the ROC
