# Supplementary figures and images for: Diagnostic performance of the normal range of gastrin calculated using strict criteria based on a combination of serum markers and pathological evaluation for detecting gastritis: a retrospective study
Source: BMC Gastroenterol. 2023 May 20;23:167. doi: 10.1186/s12876-023-02816-1 (PMC10199508; doi:10.1186/s12876-023-02816-1)

**Supplementary Fig. 1** Analysis flow

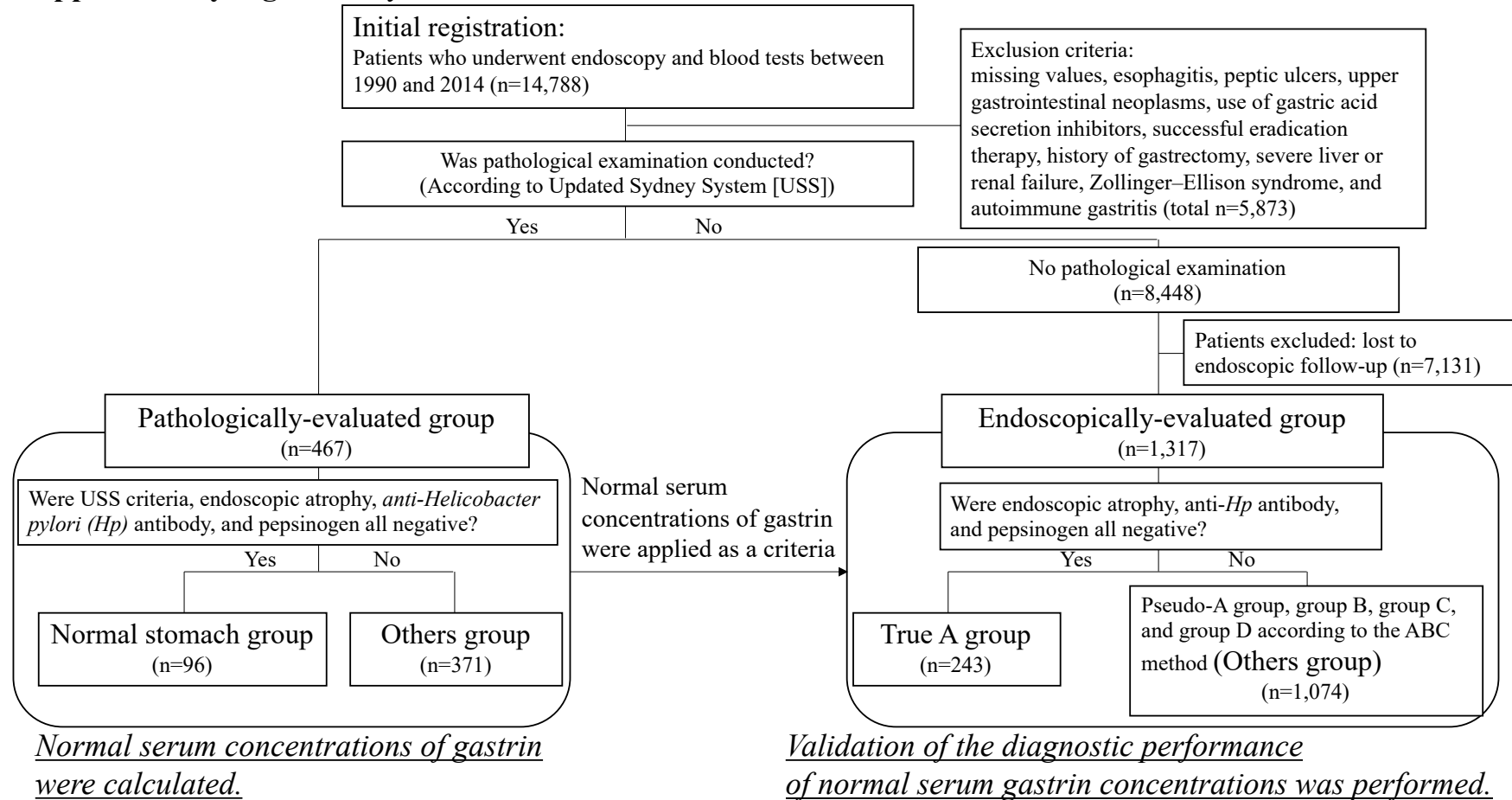

Supplement: Supplementary file 1 — Additional file 1: Supplementary Fig. 1. Analysis flow. [file 12876_2023_2816_MOESM1_ESM.pdf]
