# Supplementary material for: Diagnostic performance of the normal range of gastrin calculated using strict criteria based on a combination of serum markers and pathological evaluation for detecting gastritis: a retrospective study
Source: BMC Gastroenterol. 2023 May 20;23:167. doi: 10.1186/s12876-023-02816-1 (PMC10199508; doi:10.1186/s12876-023-02816-1)
Supplement: Supplementary file 2 — Additional file 2: Supplementary Table 1. Definitions of the pathologically- and endoscopically- evaluated groups. Supplementary table 2. Comparison between classification by the ABC methods and by Updated Sydney System for diagnosis of gastritis. [file 12876_2023_2816_MOESM2_ESM.pdf]

Supplementary Table 1. Definitions of the pathologically- and endoscopically- evaluated groups

	PG	Anti-*Hp* antibody	Endoscopic atrophy	Pathological atrophy
Pathologically normal stomach	(−)	(−)	(−)	(−)
True A	(−)	<3	(−)	N/A
Pseudo A	(−)	<3	(+)	N/A
Group B	(−)	≥3	Any	N/A
Group C	(+)	≥3	Any	N/A
Group D	(+)	<3	Any	N/A

Hp, *Helicobacter pylori*; PG, pepsinogen

Supplementary table 2. Comparison between classification by the ABC methods (anti-*Hp* antibody and PG concentration) and by Updated Sydney System for diagnosis of gastritis

Pathological marker	Updated Sydney System	
	Normal (n=96)	Non-normal (n=371)
Serum anti-*Hp* antibody (–) and PG (–) (n=131)	96	35
Serum anti-*Hp* antibody (+) or PG (+) (n=336)	0	336

Hp, *Helicobacter pylori*; PG, pepsinogen
